# Structural Features and Optical Properties of All-Inorganic Zero-Dimensional Halides Cs_4_PbBr_6–*x*_I*_x_* Obtained by Mechanochemistry

**DOI:** 10.1021/acsami.3c07707

**Published:** 2023-08-18

**Authors:** Carmen Abia, Carlos A. López, Javier Gainza, João Elias F. S. Rodrigues, Brenda Fragoso, Mateus M. Ferrer, Maria Teresa Fernández-Díaz, François Fauth, José Luis Martínez, José Antonio Alonso

**Affiliations:** † Instituto de Ciencia de Materiales de Madrid, CSIC, Cantoblanco, 28049 Madrid, Spain; ‡ Institut Laue Langevin, BP 156X, Grenoble F-38042, France; § INTEQUI, (UNSL-CONICET) and Facultad de Química, Bioquímica y Farmacia, UNSL, Almirante Brown 1455, 5700 San Luis, Argentina; ∥ CELLS−ALBA Synchrotron, Cerdanyola del Valles, Barcelona E-08290, Spain; ⊥ European Synchrotron Radiation Facility (ESRF), 38000 Grenoble Cedex, France; # CCAF, PPGCEM/CDTec, Federal University of Pelotas, 96010-610 Pelotas, Rio Grande do Sul, Brazil

**Keywords:** Cs_4_PbBr_6_, Cs_4_PbI_6_, mechanochemical synthesis, synchrotron X-ray diffraction, optoelectronic properties, tunable band gap

## Abstract

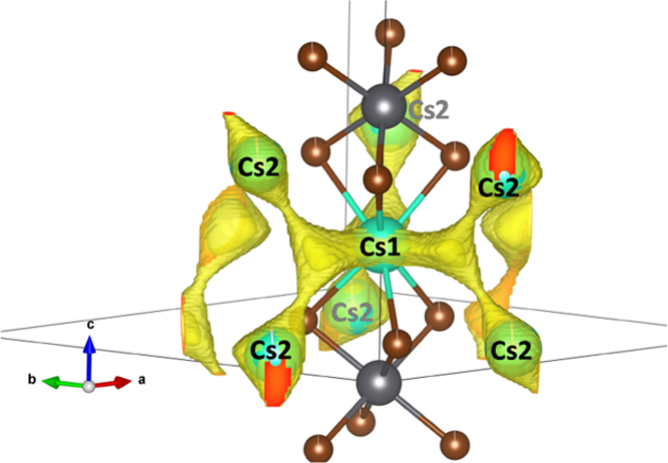

Despite the great success of hybrid CH_3_NH_3_PbI_3_ perovskite in photovoltaics, ascribed to its excellent optical absorption properties, its instability toward moisture is still an insurmountable drawback. All-inorganic perovskites are much less sensitive to humidity and have potential interest for solar cell applications. Alternative strategies have been developed to design novel materials with appealing properties, which include different topologies for the octahedral arrangements from three-dimensional (3D, e.g., CsPbBr_3_ perovskite) or two-dimensional (2D, e.g., CsPb_2_Br_5_) to zero-dimensional (0D, i.e., without connection between octahedra), as the case of Cs_4_PbX_6_ (X = Br, I) halides. The crystal structure of these materials is complex, and their thermal evolution is unexplored. In this work, we describe the synthesis of Cs_4_PbBr_6–*x*_I*_x_* (*x* = 0, 2, 4, 6) halides by mechanochemical procedures with green credentials; these specimens display excellent crystallinity enabling a detailed structural investigation from synchrotron X-ray powder diffraction (SXRD) data, essential to revisit some features in the temperature range of 90–298 K. In all this regime, the structure is defined in the trigonal *R*3̅*c* space group (#167). The presence of Cs and X vacancies suggests some ionic mobility into the crystal structure of these 0D halides. Bond valence maps (BVMs) are useful in determining isovalent surfaces for both Cs_4_PbBr_6_ and Cs_4_PbI_6_ phases, unveiling the likely ionic pathways for cesium and bromide ions and showing a full 3D connection in the bromide phase, in contrast to the iodide one. On the other hand, the evolution of the anisotropic displacement parameters is useful to evaluate the Debye temperatures, confirming that Cs atoms have more freedom to move, while Pb is more confined at its site, likely due to a higher covalency degree in Pb–X bonds than that in Cs–X bonds. Diffuse reflectance ultraviolet–visible (UV–vis) spectroscopy shows that the optical band gap can be tuned depending on iodine content (*x*) in the range of 3.6–3.06 eV. From density functional theory (DFT) simulations, the general trend of reducing the band gap when Br is replaced by I is well reproduced.

## 1 Introduction

Solar energy presents a viable solution to the pressing issue of global warming induced by CO_2_ emission from conventional sources, such as coal and fossil fuels.^1^ It can be converted into electric energy in solar cells, which still pose interesting challenges. Although single-crystalline materials like silicon and other semiconductor compounds^2^ have yielded excellent efficiencies, their manufacturing processes remain expensive. In contrast, organic and dye-sensitized solar cells offer a more cost-effective technology using solution-processed materials. However, these cells must achieve further enhancements in power conversion efficiency (PCE) and ensure long-term operation without the degradation of the active materials to be viable for industrial application. Therefore, efforts to reduce fabrication costs and increase PCE must walk hand in hand with long-term material stability considerations.

In the past decade, there has been a remarkable increase in the photoelectric conversion efficiency (PCE) of halide perovskite photovoltaic devices, with a record-high value of around 25.6% on black-phase formamidinium (FA = CH(NH_2_)_2_) lead iodide (α-FAPbI_3_).^3^ Recent reports have also demonstrated highly efficient photovoltaic devices with certified power conversion efficiencies approaching 20% in methylammonium (MA) lead halides MAPbX_3_ (MA = CH_3_NH_3_; X = Br, Cl, and I) as semiconducting absorber layers, which is highly encouraging.^4−8^ However, MAPb(Br*_x_*I_1–*x*_)_3_ hybrid solar cells exhibited serious PCE degradation after exposure to humidity,^9^ which remains an unresolved problem.

A closely related family of compounds is the all-inorganic cesium lead halide perovskites (CsPbX_3_, X = Br, Cl, I, and mixed Cl/Br and Br/I), which are isostructural to orthorhombic perovskite CaTiO_3_ and related oxides.^10−13^ These ternary compounds are by far less soluble in common solvents, contrary to MAPbX_3_, and therefore much more stable under humid and harsh conditions. The photoconductivity and direct band gap of CsPbX_3_ were reported more than 50 years ago,^14^ but many aspects remain unexplored including the phase transition mechanisms and charge-transfer process.^15^

Earlier studies were focused on the three-dimensional (3D) CsPbX_3_ perovskites with Cs^+^ ions filling the voids created by eight neighboring corner-sharing [PbX_6_]^4–^ octahedra.^16−20^ Reducing the dimensionality in layered halide perovskites (LHPs) has been a successful strategy for tailoring novel properties and applications, such as in two-dimensional (2D) ″Ruddlesden–Popper″ A_2_PbX_4_ phases.^21−25^ These structures consist of layers of corner-sharing [PbX_6_]^4–^ octahedra alternating with layers of bulky cations, achieved using a mixture of smaller and larger (e.g., butylammonium) monovalent cations.^26^ In these 2D structures, the exciton can no longer propagate in all dimensions and is instead confined in two-dimensional [PbX_6_] layers that result in larger band gaps than the ABX_3_ phase.

The A_4_PbX_6_-type structure (A = Rb^+^, Cs^+^) features [PbX_6_]^4–^ octahedra that are completely decoupled in all dimensions,^27−30^ resulting in elevated insulator band gaps (Cs_4_PbCl_6_ = 4.37 eV, Cs_4_PbBr_6_ = 3.95 eV, and Cs_4_PbI_6_ = 3.38 eV). This fact makes the A_4_PbX_6_ phase often referred to as a zero-dimensional (0D) perovskite.^31−33^ The optical properties of such crystals closely resemble those of individual [PbX_6_]^4–^ clusters observed experimentally in halide salts doped with Pb^2+^ ions. While the 3D and 2D phases of LHPs are well studied and reported, the 0D perovskites are comparatively less explored, with only very recent works revisiting the optical properties of Cs_4_PbBr_6_ nanocrystals.^34−37^

In this work, we report on a fast and straightforward ambient synthesis method for Cs_4_PbBr_6–*x*_I*_x_* (*x* = 0, 2, 4, 6) solid solution by mechanochemical milling in an inert atmosphere. The as-prepared samples were investigated using high-angular resolution synchrotron X-ray diffraction (SXRD) and the optical characterization from ultraviolet–visible (UV–vis) spectroscopy. The optical studies have demonstrated that the optical band gap can be tuned depending on iodine content (*x*) in the range of 3.6–3.06 eV. From the Rietveld refinement of the SXRD data, the ionic path mobility was determined from bond valence maps (BVMs). In addition, we estimated the Debye temperatures for the atomic displacement factors, yielding interesting trends for the relative strength of Cs–X versus Pb–X chemical bonds, which were further corroborated by density functional theory (DFT) calculations.

## 2 Experimental Methods

### 2.1 Sample Synthesis

The Cs_4_PbBr_6–*x*_I*_x_* (*x* = 0, 2, 4, 6) solid solution was synthesized by the mechanochemical method at room temperature.^38,39^ Stoichiometric amounts of CsBr, CsI, PbBr_2_, and/or PbI_2_ were milled in a N_2_ atmosphere using a planetary ball mill. A total of 1.5 g of reactants were milled using 30 zirconia balls of 5 mm diameter, with a final 8.3:1 mass ratio for 4 h at 450 rpm in a Retsch PM100 mill. The phase formation was evaluated by the laboratory X-ray diffraction (XRD) patterns collected on a Bruker D5 diffractometer with a Cu Kα (λ = 1.5418 Å) radiation source.

### 2.2 Synchrotron X-ray Diffraction and Analysis

The SXRD patterns were collected with a high-angular resolution setup (MAD) in the BL04-MSPD beamline at ALBA Synchrotron Light Source in Barcelona (Spain).^40^ The powder samples were contained in 0.3 mm diameter quartz capillaries to avoid absorption effects. An incident beam of 38 keV in energy (λ = 0.3262 Å) was selected by Si(111) crystals, and the beam spot size was collimated by slits to 3(H) × 1(V) mm^2^ final dimensions. During the SXRD data collection, the capillaries were put in rotation mode for avoiding preferential orientation. The absorption effect was considered during the refinements by considering the μR coefficients estimated theoretically for the X-ray energy, capillary diameter (0.15 mm), and packing factor (0.5). For Cs_4_PbBr_6_ and Cs_4_PbI_6_, μR coefficients are, respectively, 2.52 and 4.06. The temperature-dependent measurements were performed using an Oxford cryostream cooler air gun (N_2_ gas pump) to reach the following temperatures: 90, 120, 150, 200, 250, and 298 K. The diffraction patterns were refined by the Rietveld method in the framework of FullProf software.^41,42^ The background was interpolated between regions devoid of reflections, and the pseudo-Voigt profile function was chosen to generate the line shape of the diffraction peaks. Then, the following parameters were refined in the final run: scale factor, background coefficients, zero-point error, pseudo-Voigt corrected for the asymmetry parameters, positional coordinates, anisotropic displacement factors (*U*^ij^), and occupancy factors (*f*_occ_).

### 2.3 Optical Properties and Analysis

The optical diffuse reflectance spectra of the powder halides were measured at ambient conditions using a UV–vis spectrophotometer Varian Cary 5000. A diffuse reflectance cell was employed with an integrating sphere for estimating the absorption coefficient (α). Such a coefficient can be determined by the Kubelka–Munk function [*F*(*R*)].^43^ The band gap energy was then estimated with the spectrophotometer from the diffuse reflectance data fitted by the equation [*h*ν*F*(*R*)]^1/*n*^ = *A*(*h*ν – *E*_G_). The exponent *n* depends on the nature of the electron transition: 1/2 or 2 for direct or indirect transition gaps, respectively.

## 3 Computational Methods

The models based on density functional theory (DFT) were built to understand the electronic transitions and the chemical environment of Cs_4_PbX_6_ (X = Br, I) halides. These models were created using the CRYSTAL17 package with the PBE functional.^44,45^ The atomic bases were the triple-zeta valence with the polarization Gaussian basis (POB-TZVP) sets developed by Laun et al.^46^ The Coulomb and exchange series thresholds are set according to five parameters, namely, overlap and penetration for Coulomb integrals, the overlap for HF exchange integrals, and the pseudo-overlap, being set as 8, 8, 8, 8, and 16, respectively. The shirking factors (Pack–Monkhorst and Gilat net) were 6 and 6, respectively. Both the gradient component and the nuclear displacement were chosen with a tolerance on their root mean square of 0.0003 and 0.0012 arb. units, respectively. The topological analysis of the critical points of the chemical bonds was performed according to the “quantum theory: atoms in molecules” (QTAIM) with TOPOND software.

## 4 Results

### 4.1 Initial Characterization

**Figure 1:**
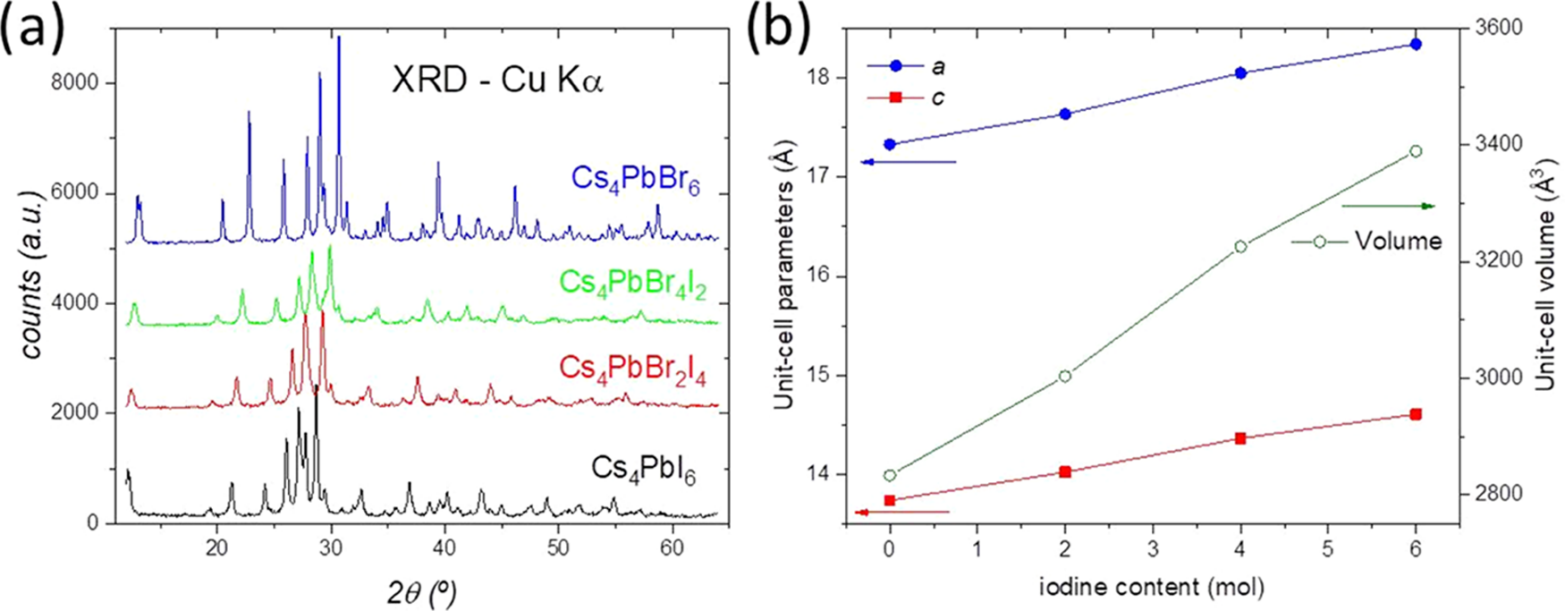
Laboratory X-ray powder diffraction patterns of Cs_4_PbBr_6–*x*_I*_x_* (a). Variation of the unit cell parameters (*a*, *b*, and *V*) with iodine content (*x*) (b).

The well-crystallized samples of Cs_4_PbBr_6–*x*_I_x_ (*x* = 0, 2, 4, 6) solid solution were obtained as colored microcrystalline powders, spanning from yellow for the Br specimen to orange for the I compound. The laboratory X-ray diffraction measurements of the obtained phases are shown in Figure 1a. These patterns exhibited high purity of the phases that were indexed with the trigonal symmetry described in previous reports.^47^ The pattern indexing leads to the unit cell parameters that are plotted in Figure 1b, determined by using the Le Bail method. The iodine incorporation in the Cs_4_PbBr_6_ lattice leads to the expected expansion of the trigonal unit cell volume in a quasi-linear trend with the iodine content ranging from 0 to 6.

### 4.2 Synchrotron Structural Analysis

**Figure 2:**
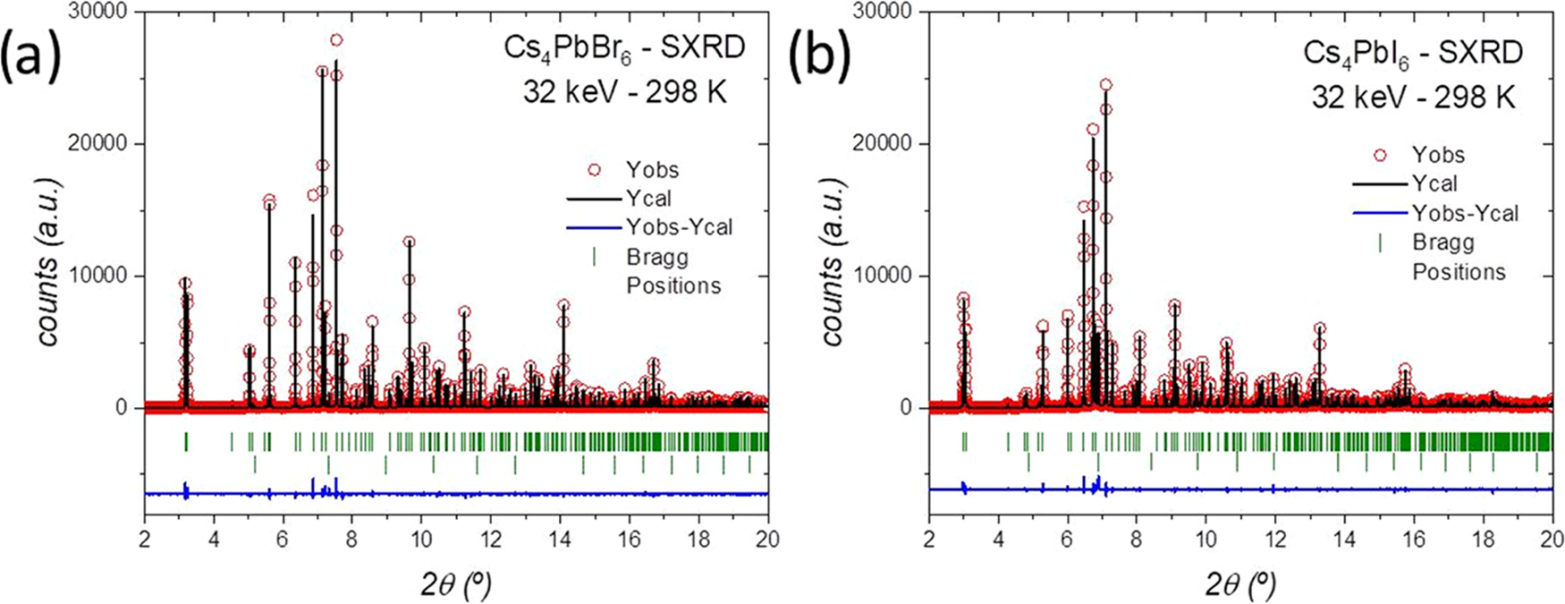
Rietveld refinements from synchrotron XRD at room temperature for (a) Cs_4_PbBr_6_ and (b) Cs_4_PbI_6_.

A deeper crystallographic analysis of the end members of the solid solution (Cs_4_PbBr_6_ and Cs_4_PbI_6_) was conducted using synchrotron X-ray diffraction and Rietveld refinements. This analysis confirmed that both phases crystallized in the trigonal *R*3̅*c* space group (#167). In this crystal structure, Pb cations are located at the 6*b* (0, 0, 0) Wyckoff site and the bromides are in 36*f* positions (*x*, *y*, *z*). The cesium cations are distributed in both 6*a* (0, 0, 1/4) and 18*e* (*x*, 0, 1/4) named Cs1 and Cs2, respectively. In these structures, the [PbX_6_] octahedra do not share corners, edges, or faces; for this reason, they are commonly named zero-dimensional perovskites. However, these structures are not strictly perovskite-type halides, as it is well known in crystallochemistry. The Rietveld refinements of the SXRD patterns can be appreciated in Figure 2 for both bromine and iodine phases. The superior quality of the patterns, resulting from the high crystallinity of the samples, allows for obtaining valuable structural information, such as the anisotropic displacement factors (*U*^ij^) for all of the atoms.

Another structural feature found by the Rietveld analysis was the presence of vacancies that were postulated in previous works.^48−50^ The final crystallographic results are listed in Tables 1 and 2 for Cs_4_PbBr_6_ and Cs_4_PbI_6_, respectively. Minor amounts of CsX were identified and included in the refinement. The crystallographic formulae for both halide phases can be written as Cs_3.933(8)_PbBr_5.933(8)_ and Cs_3.93(1)_PbI_5.93(1)_.

**Table 1:** Crystallographic Data for the Cs_4_PbBr_6_ Halide from SXRD Data at 298 K, Defined in the Trigonal *R*3̅*c* Space Group^a^

	*x*	*y*	*z*	*U*_eq_ (Å^2^)		*f*_occ_
Cs1	0	0	0.25	0.054(1)		1
Cs2	0.37524(8)	0	0.25	0.0385(8)		0.978(3)
Pb	0	0	0	0.0238(7)		1
Br	0.19255(9)	0.0291(1)	0.10135(6)	0.0372(9)		0.989(1)
atomic displacement parameters (Å^2^)						
	*U*^11^	*U*^22^	*U*^33^	*U*^12^	*U*^13^	*U*^23^
Cs1	0.067(2)	0.067(2)	0.029(1)	0.033(2)	0	0
Cs2	0.0394(8)	0.0337(8)	0.0426(8)	0.0168(8)	–0.0029(3)	–0.0058(3)
Pb	0.0241(7)	0.0241(7)	0.0231(7)	0.0121(7)	0	0
Br	0.0353(9)	0.0405(9)	0.0359(9)	0.0213(7)	–0.0050(7)	–0.0016(6)
*R*_p_ = 7.22%, *R*_wp_ = 9.68%, χ^2^ = 1.17, *R*_Bragg_ = 3.16%						
impurity: CsBr 0.8% w/w.						

a *a* = 13.71881(2) Å, *c* = 17.30779(3) Å, and *V* = 2821.01(1) Å^3^. Refined formula: Cs_3.933(8)_PbBr_5.933(8)_.

**Table 2:** Crystallographic Data for the Cs_4_PbI_6_ Phase from SXRD Data at 298 K, Defined in the Trigonal *R*3̅*c* Space Group^a^

	*x*	*y*	*z*	*U*_eq_ (Å^2^)		*f*_occ_
Cs1	0	0	0.25	0.058(2)		1
Cs2	0.3799(1)	0	0.25	0.038(1)		0.975(3)
Pb	0	0	0	0.021(1)		1
I	0.19500(8)	0.03190(9)	0.10223(5)	0.0429(9)		0.988(2)
atomic displacement parameters (Å^2^)						
	*U*^11^	*U*^22^	*U*^33^	*U*^12^	*U*^13^	*U*^23^
Cs1	0.073(2)	0.073(2)	0.029(2)	0.036(2)	0	0
Cs2	0.039(1)	0.033(1)	0.043(1)	0.017(1)	–0.0037(5)	–0.0075(5)
Pb	0.021(1)	0.021(1)	0.0190(1)	0.011(1)	0	0
I	0.041(1)	0.0469(9)	0.0408(9)	0.0252(8)	–0.0056(8)	–0.0015(7)
*R*_p_ = 7.00%, *R*_wp_ = 9.48%, χ^2^ = 1.40, *R*_Bragg_ = 3.45%						
Impurity: CsI 4.6% w/w.						

a *a* = 14.56746(4) Å, *c* = 18.31058(5) Å, and *V* = 3365.12(2) Å^3^. Refined formula: Cs_3.93(1)_PbI_5.93(1)_.

**Figure 3:**
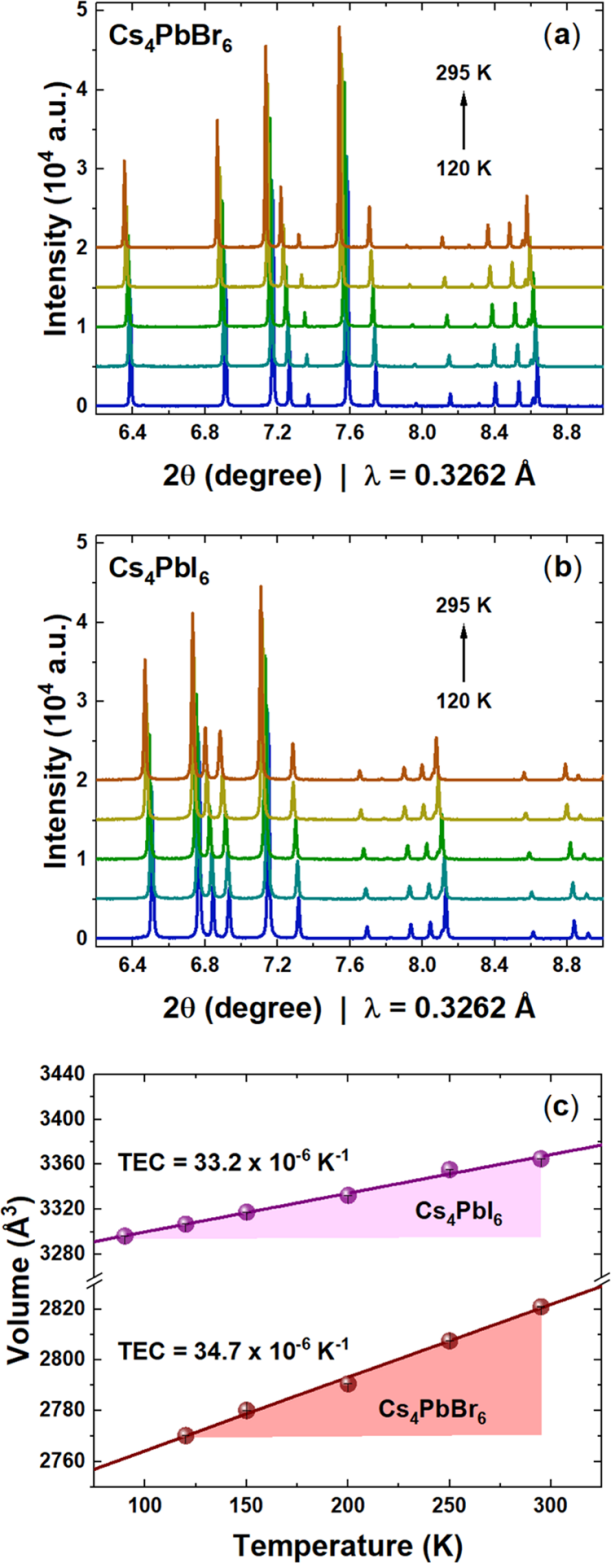
Temperature dependence of the SXRD patterns (2θ range of 6.2–9.0°) of Cs_4_PbBr_6_ (a) and Cs_4_PbI_6_ (b). Thermal expansion of the unit-cell volume from which the coefficients of thermal expansion (TEC) are estimated (c).

We also probed the temperature dependence of the crystal structure in the range of 120–295 K. Figure 3a,b exhibits the low-temperature synchrotron XRD patterns of both Cs_4_PbBr_6_ and Cs_4_PbI_6_ halides, respectively, elucidating that no phase transition takes place, and therefore, the *R*3̅*c* unit cell is maintained in this temperature range. In Figure 3c, the coefficient of thermal expansion (TEC) of the unit cell volume was estimated from a linear extrapolation of the volume versus temperature curve. The obtained coefficients are 34.7 × 10^–6^ and 33.2 × 10^–6^ K^–1^ for Cs_4_PbBr_6_ and Cs_4_PbI_6_, respectively.

### 4.3 Optical Properties

**Figure 4:**
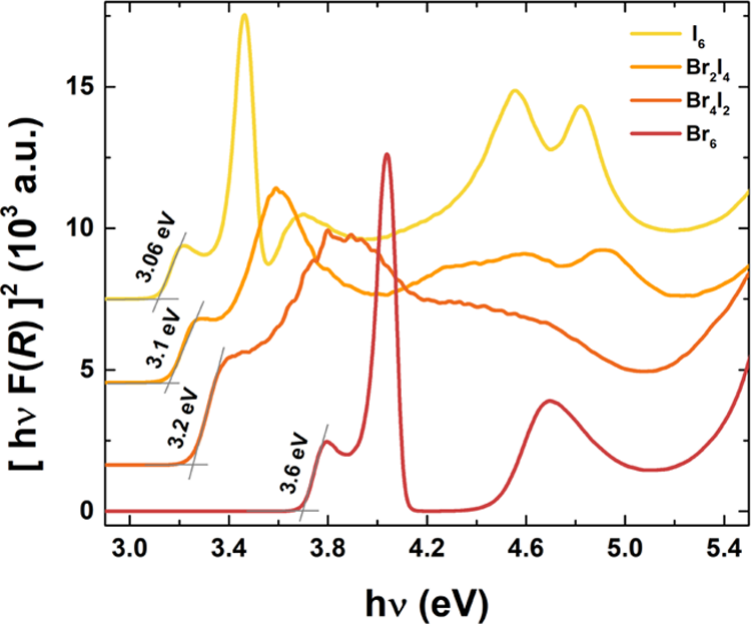
Room temperature UV–vis spectra for the zero-dimensional halide perovskite Cs_4_PbBr_6–*x*_I*_x_* (*x* = 0, 2, 4, 6). The spectra were vertically shifted to clarify the representation.

The absorption capacity of Cs_4_PbBr_6–*x*_I*_x_* (*x* = 0, 2, 4, 6) perovskite powders were investigated by diffuse reflectance UV–vis spectroscopy. Figure 4 shows the optical absorption coefficient related to the Kubelka–Munk function *F*(*R*) = (1 – *R*)^2^/2*R* (where *R* is the reflectance of each sample) versus wavelength in units of eV. The direct band gap for each perovskite has been calculated by extrapolating the linear region to the abscissa (see in the Experimental Methods section). The value obtained for Cs_4_PbBr_6_ is around 3.6 eV. As expected, a red shift is observed for the perovskite Cs_4_PbI_6_ (where one Br atom has been replaced by iodine), with a band gap of 3.06 eV. The general behavior of the band gap as a function of iodine content denotes that this parameter can be tunable for providing a particular gap value.

## 5 Discussion

### 5.1 Defect Structures and Ionic Mobility in 0D Halide Perovskites

Rietveld analysis revealed the presence of bromide and iodine vacancies that were postulated in previous works. Indeed, the superior luminescent properties were better understood considering the vacancy level of bromine anions.^48−50^ To contribute crystallographic evidence for this hypothesis, the vacancies in both crystal structures were examined carefully. Initially, the occupancy factors were refined individually to find the vacancies in each site. These initial analyses reveal occupancies lower than 1 in Cs2 and in the halide site for both Cs_4_PbBr_6_ and Cs_4_PbI_6_ phases. Cs1 exhibited a negligible deficiency, and its occupancy was fixed to unity (see Tables 1 and 2). Considering that the vacancy levels of halide and Cs are similar, it is possible to deduce the concomitant nature in both defects that correspond to the crystal electroneutrality. This fact suggests that these materials present Schottky defects, as given by the Kröger–Vink notation
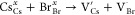
1
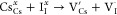
2for both Cs_4_PbBr_6_ and Cs_4_PbI_6_, respectively.

**Figure 5:**
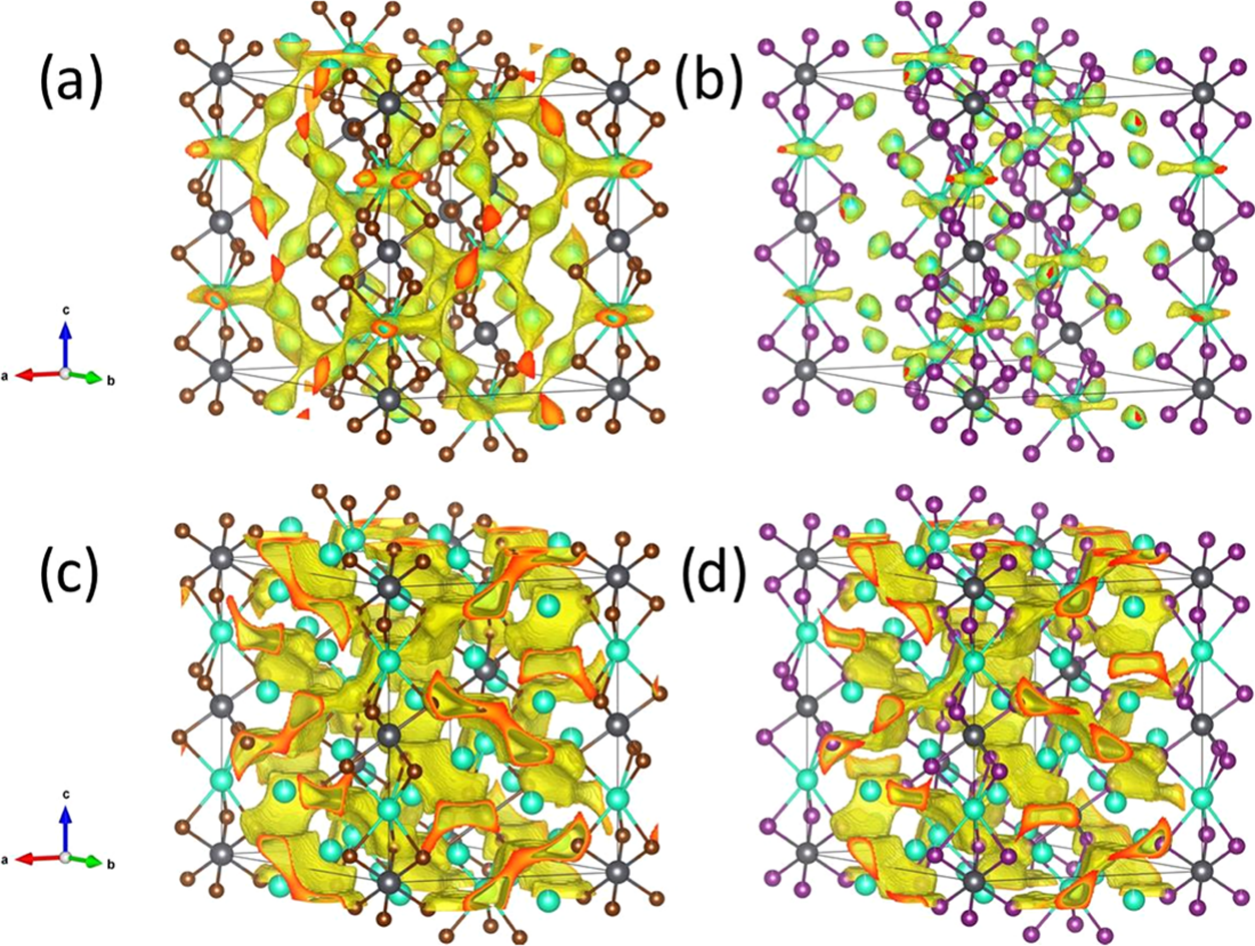
Isovalent surface obtained from bond valence maps for Cs_4_PbBr_6_ (a) Cs^+^ and (c) Br^–^ and for Cs_4_PbI_6_ (b) Cs^+^ and (d) I^–^. Green, gray, brown, and purple spheres represent the cesium, lead, bromide, and iodine atoms, respectively.

In the final refinement run, the occupancy factors of halide and Cs2 atoms were simultaneously refined. Overall, there is not much difference in the vacancy level in this refinement, and finally, in both phases, the occupancies were quantified as ∼1% of halide anions. Such a crystallographic feature allows us to predict some ionic (both anionic and cationic) mobility into the crystal structure of the 0D halides. To deepen into that, the possible ionic paths were analyzed by bond valence methods.^51^ The bond valence maps (BVMs) were calculated for both Cs^+^ and Br^–^ (and I^–^) at room temperature. Figure 5 illustrates the isovalent surfaces for both Cs_4_PbBr_6_ and Cs_4_PbI_6_ phases, revealing the likely ionic pathways for cesium and halide ions.

**Figure 6:**
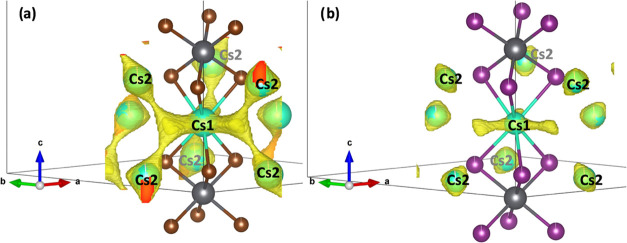
Isovalent surface from bond valence maps around Cs^+^ atoms demonstrating the likely jump possibilities in 0D: (a) Cs_4_PbBr_6_ and (b) Cs_4_PbI_6_. Green, gray, and brown spheres represent the cesium, lead, and bromide atoms, respectively.

Cs^+^ isovalent maps (Figure 5a,b) show a full 3D connection in the bromine phase, in contrast to the iodine one. This fact suggests that Cs^+^ has more freedom to move in the lattice only in Cs_4_PbBr_6_ than the iodine counterpart. Cs^+^ presents 3D mobility, and each Cs1 can jump to another six Cs2 positions (see the density around the green spheres). These jumps are not linear: first, they start with a threefold displacement in the *ab* plane like a triangle, and then from these three sides, the Cs^+^ can jump up or down toward the adjacent Cs2 position (in Figure 6a). In contrast, Cs2 has only two possibilities to jump to the Cs1 site that acts as an intermediate position between two Cs1 sites in different *ab* planes. This situation is different in Cs_4_PbI_6_ (Figure 6b), where the isosurface displays a threefold displacement of Cs1 but it is not connected to Cs2.

The bond valence maps also displayed the possibility of anionic mobility (see Figure 5c,d). These maps demonstrated that the halides could move in channels along three directions [0 1 −1], [1 0 1], and [−1 1 1]. Figure 5a,b elucidates the views of the X^–^ isovalent surface along the [0 1 −1] direction for the bromine and iodine phases, respectively. These channels can also be seen in the density planes (0 1 −1) in Figure 5c,d. Additional isovalent surfaces and planes are illustrated in Figures S1 and S2 in the Supporting Information. These pathways are along the *a, b*, and *c* directions of the rhombohedral unit cell (*a*_R_ ∼9.80 Å; α_R_ ∼88.86° and *a*_R_ ∼10.39 Å; α_R_ ∼89.00° for Cs_4_PbBr_6_ and Cs_4_PbI_6_, respectively). Therefore, we may propose that the anion mobility occurs in one-dimensional channels along three possible directions at an inclination close to ∼89°.

Regarding potential and percolation energies, these parameters were calculated for Cs^+^ and X^–^ from the bond valence energy landscape (BVEL).^52^ The percolation energies and the volume fraction for ion mobility are listed in Table 3. Although these values cannot be directly related to the band gap energy obtained from ab initio calculations or other experimental studies, these results allow some predictions about ionic conductivity. The superior volume mobility for bromine suggests that the ionic conductivities for Cs^+^ and X^–^ are higher in Cs_4_PbBr_6_, as confirmed by the lower percolation energy (Cs^+^: 0.09–0.10 eV; X^–^: 0.57 eV). For cesium cations, the percolation energy for Cs_4_PbBr_6_ is almost half of the values obtained in the iodine phase, which agrees with the absence of percolation pathways in the BVM, as shown in Figure 5b. For the halide conduction, the lower energy for the bromine phase suggests a higher mobility in Cs_4_PbBr_6_ with a percolation energy of around 0.57 eV than that in Cs_4_PbI_6_ with 0.84–0.85 eV.

**Table 3:** Percolation Energy and Volume mobility fraction for Cs_4_PbBr_6_ and Cs_4_PbI_6_

ion	direction	Cs_4_PbBr_6_	Cs_4_PbI_6_
Cs^+^	*a*-axis	0.09 eV	0.23 eV
	*c*-axis	0.10 eV	0.23 eV
	volume mobility	6.0%	3.0%
X^–^	*a*-axis	0.57 eV	0.84 eV
	*c*-axis	0.57 eV	0.85 eV
	volume mobility	2.6%	1.6%

### 5.2 Bond Stiffness from the Harmonic Debye Analysis

The high quality of synchrotron data was suitable to probe the thermal variation of the mean-square displacement factors (MSDs, in units of Å^2^) as a function of temperature. These parameters were analyzed in their equivalent displacement factors (*U*_eq_), as derived from the independent anisotropic coefficients *U*^11^, *U*^22^, *U*^33^, *U*^12^, *U*^13^, and *U*^23^ for each atom within the trigonal *R*3̅*c* crystal structure. The temperature evolution of the equivalent displacements for each atom can be described in the framework of the harmonic Debye model,^53,54^ as summarized by the following equation
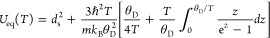
3where θ_D_ represents the Debye temperature, *m* is the atomic mass, *d*_*s*_^2^ is the intrinsic disorder parameter that accounts for the 0 Kelvin vibration (i.e., ground-state), *T* is the absolute temperature (in units of Kelvin), and *k*_B_ and ℏ are the Boltzmann and reduced Planck constants, respectively.

**Figure 7:**
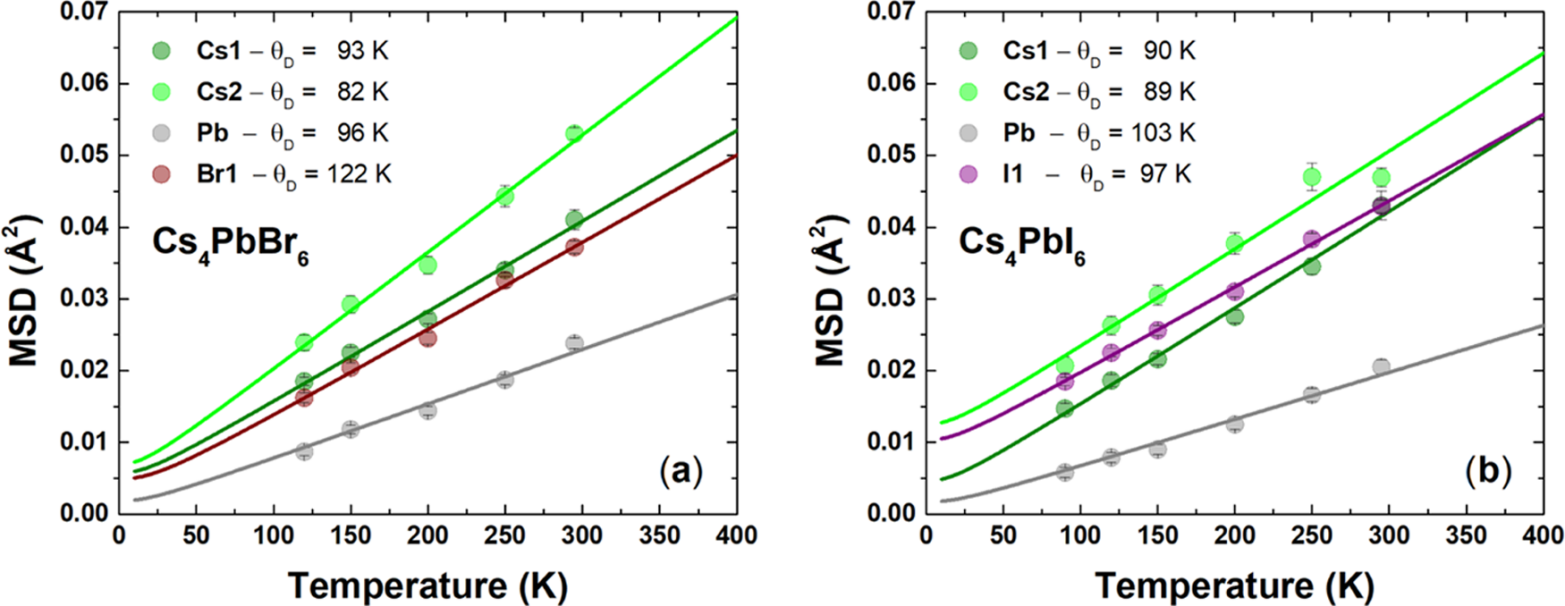
Thermal evolution of the MSDs (*U*_eq_) of Cs1, Cs2, Pb, Br, and I for Cs_4_PbBr_6_ (a) and Cs_4_PbI_6_ (b). Circles represent the MSDs extracted from the Rietveld refinement, while the lines are the best fits to the experimental data using the harmonic Debye model.

Figure 7 exhibits the best fittings of the MSDs using the harmonic Debye model for the Cs_4_PbBr_6_ (a) and Cs_4_PbI_6_ (b) phases. The nonlinear least squares regression allowed for obtaining both Debye temperature and intrinsic disorder for Cs1, Cs2, and Br1, while only the θ_D_ value was estimated for Pb. In this case, *d*_*s*_^2^ was kept constant and equal to 0 because the fittings always provided values slightly below zero and, therefore, were not reliable. Table 4 lists all of the best-fitted parameters from the Debye model. In general, the Debye temperature has lower values for Cs1 and Cs2 in both halides, which means that cesium can vibrate more freely than lead and halide atoms. The θ_*D*_ value of Pb increased from 96(1) to 103(1) K when Br is replaced by I, meaning that lead atomic motion is now more restricted because of iodine atoms. Instead, the Debye temperature of the halide site decreased from 122(1) to 97(1) K; however, the *d*_s_^2^ parameter varied from 0.001(1) to 0.007(3) Å^2^ for Br and I, respectively, which points out for a more disordered halide site for the iodine-based sample.

**Table 4:** Fitted Parameters (θ_D_ and *d*_s_^2^) from the Harmonic Debye Model and the Force Constant (*k*_OPP_) Estimated from the One-Particle Harmonic Potential (OPP) Model

	Cs_4_PbBr_6_			Cs_4_PbI_6_		
	θ_D_ (K)	*d*_s_^2^ (Å^2^)	*k*_OPP_ (eV/Å^2^)	θ_D_ (K)	*d*_s_^2^ (Å^2^)	*k*_OPP_ (eV/Å^2^)
Cs1	93(1)	0.002(8)	0.68	90(1)	0.001(6)	0.64
Cs2	82(1)	0.003(6)	0.53	89(1)	0.009(4)	0.63
Pb	96(1)	0.0	1.13	103(1)	0.0	1.31
X	122(1)	0.001(1)	0.71	97(1)	0.007(3)	0.71

The bond stiffness can be estimated from the Debye temperature by the harmonic one-particle potential model (OPP), which provides the force constant of the potential as follows^55,56^
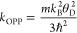
4where *k*_OPP_ represents the force constant in units of eV/Å^2^ (see Table 4). In general, the force constants for lead are almost twice higher than those for cesium in both Cs_4_PbBr_6_ and Cs_4_PbI_6_ halides. This result confirms the previous conclusion that the Cs atom has more freedom to move, while Pb is more confined at its site. It indicates that lead has more rigid motions than cesium, likely due to a higher covalency degree in Pb–X bonds than that in Cs–X bonds. The covalency of the Pb–X bonds also increased because of the replacement Br → I. In fact, *k*_OPP_ became stiffer by varying from 1.13 to 1.31 eV/Å^2^, i.e., being therefore more localized at the level of [PbX_6_] octahedron.

To better understand the bond covalency, we have estimated selected critical parameters for the bonds in both Cs_4_PbBr_6_ and Cs_4_PbI_6_ halides, namely, Pb–X and Cs–X. These parameters concern the electron density (ρ), Laplacian of the electron density (∇^2^ρ), virial field density (ϰ), Lagrangian kinetic energy density (*G*), and the total energy (*H*) of the pair bonds, as summarized in Table 5. The higher ρ values for Pb–X (29.6 × 10^–3^) bonds than those for Cs–X (6.4–10.8 × 10^–3^) confirmed that the charge density is more localized around the lead atoms. The positive values of ∇^2^ρ can be attributed to an ionic character for all of the pair bonds. The magnitude of the total energy (*H*) has opposite signals for Pb–X and Cs–X pairs, which is also reflected through the ratio |ϰ|/*H*. Here, an almost 1-fold magnitude difference seen in Pb–X and Cs–X strongly suggests that Pb–X can have a transient behavior and that a covalent character cannot be neglected.^57,58^

**Table 5:** Critical Point Parameters from Topochemical Analysis Using the Theoretical Structural Model from DFT Calculations: Bond Length, Electron Density (ρ), Laplacian of the Electron Density (∇^2^ρ), Virial Field Density (ϰ), Lagrangian Kinetic Energy Density (*G*), and the Total Energy (*H*) of the Bonds

bond	length (Å)	ρ (×10^–3^)	∇^2^ρ (×10^–2^)	*G* (×10^–3^)	*H* (×10^–3^)	ϰ (×10^–3^)	|ϰ/|H
Cs_4_PbBr_6_							
Pb–Br	3.06	29.6	5.87	16.3	–1.67	–0.18	–0.11
Cs–Br	3.69	9.73	3.12	7.30	0.49	6.80	13.88
Cs–Br	3.71	9.60	2.39	5.41	0.56	4.85	8.66
Cs–Br	3.82	7.68	1.94	4.27	0.58	3.69	6.36
Cs_4_PbI_6_							
Pb–I	3.26	29.6	4.24	12.2	–1.58	–0.14	–0.09
Cs–I	3.78	10.8	2.44	5.71	0.39	5.32	13.64
Cs–I	3.91	8.35	1.90	4.30	0.46	3.84	8.35
Cs–I	4.03	6.45	1.53	3.31	0.52	2.80	5.38

### 5.3 Effect of Dimensionality on the Band Gap Energy

**Figure 8:**
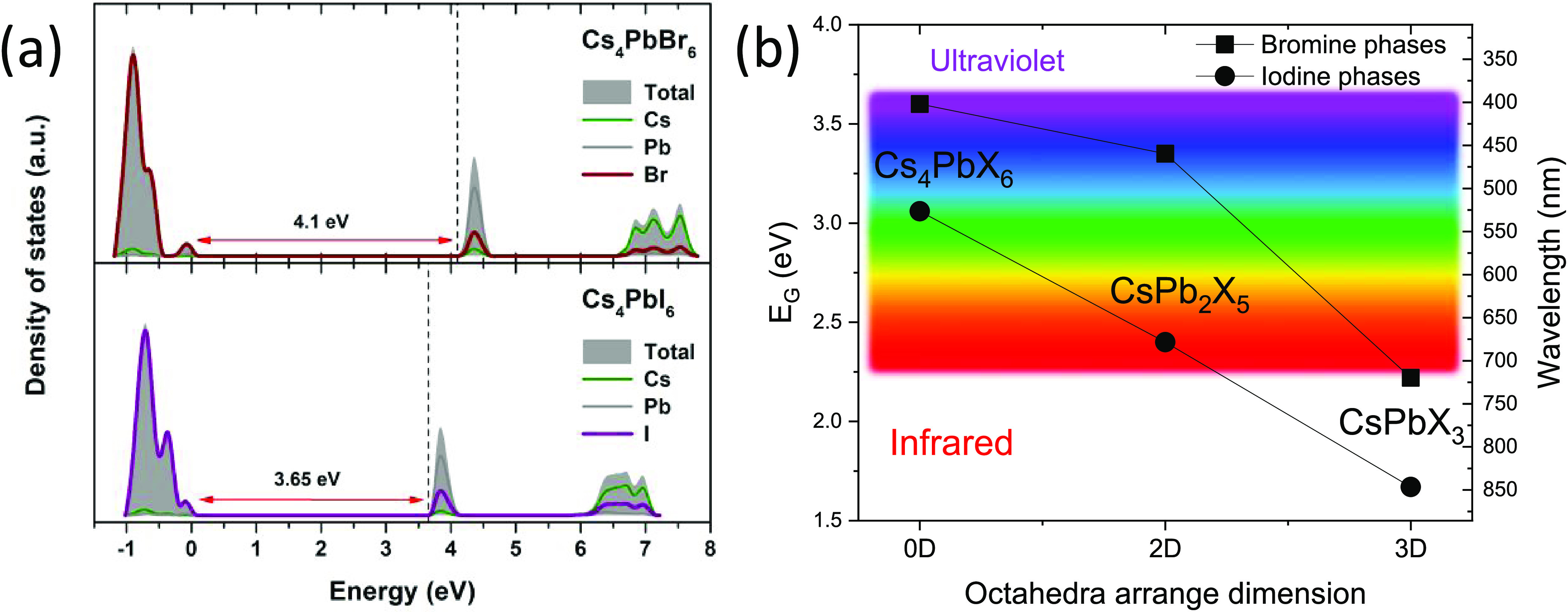
Partial density of states (PDOS) for Cs_4_PbX_6_ (X = Br, I) halides (a). Comparison of the band gap energy (*E*_G_) values for the Cs–Pb–X (X = Br, I) phases with different octahedral arrangement dimensions (b).

From the DFT simulation, we obtained the partial density of states as represented in Figure 8a. The transition was confirmed to be a direct one (see Figure S3 in the Supporting Information). The calculations overestimated the band gap energies for both halides, such that *E*_G_ values are 4.1 eV (expt. 3.6 eV) and 3.65 eV (expt. 3.06 eV) for X = Br and I, respectively. However, the general trend of reducing the band gap when Br is replaced by I was reproduced. Indeed, iodine reduces the band gap by a factor of Δ*E*_G_ = 0.54 eV (DFT. Δ*E*_G_ = 0.45 eV), enabling a band gap optimization from ultraviolet down to green in the visible spectrum. Figure 8b summarizes the band gap energy (*E*_G_) of the 0D Cs_4_PbX_6_ (X = Br, I) phases compared with CsPb_2_X_5_^59,60^ and CsPbX_3_^61,62^ phases that possess 2D and 3D octahedral arrangements, respectively. This plot allows a comparison of the band gap values with dimensionality (0D, 2D, or 3D). The 0D family shows a hypsochromic shift (change to shorter wavelength or higher frequency), with respect to 2D and 3D Cs–Pb halide phases. From this comparison, we can conclude that the optical band gap of Cs–Pb halides is tuned by the nature of the halide atom (Br, Cl, or I), and dimensionality plays a role that needs to be considered for further improvements of the optical device performances of these compounds.

## 6 Conclusions

Well-crystallized Cs_4_PbX_6_ (X = Br, I) halides were synthesized by a mechanochemical method under mild milling conditions. The crystallographic features were analyzed from synchrotron X-ray powder diffraction in the 90–298 K temperature range, where the crystal structure is trigonal *R*3̅*c* and contains isolated [PbX_6_] octahedra corresponding to these 0D perovskites. The lattice parameters and volume linearly increase with temperature. From the mean-square displacement factors, the bonding stiffness was estimated using the harmonic one-particle potential (OPP), showing that the Pb–X bonds are stiffer than the Cs–X bonds. Moreover, bond valence maps suggest the possibility of both cationic and anionic mobility in channels along the three directions [0 1 −1], [1 0 1], and [−1 1 1]. DFT calculations determined higher electron density values for Pb–X (ρ = 29.6 × 10^–3^) bonds than those for Cs–X (ρ = 6.4–10.8 × 10^–3^), confirming that the charge density is more localized around lead atoms. The direct band gap for each perovskite, determined by diffuse reflectance UV–vis spectroscopy, yields a value of around 3.6 eV for Cs_4_PbBr_6_; a red shift is observed as I content increases in Cs_4_PbBr_6–*x*_I*_x_* with a band gap of 3.06 eV for *x* = 6. Indeed, iodine reduces the band gap by a factor of Δ*E*_G_ = 0.54 eV (calculated by DFT, Δ*E*_G_ = 0.45 eV), enabling a band gap optimization from ultraviolet down to green in the visible spectrum. The optical band gap of Cs–Pb halides is tuned by the halide atom, and dimensionality needs to be considered for further improvements in the optical device performances of these compounds.

## Supporting Information Available

Additional isovalent surfaces for Cs_4_PbBr_6_, as well as the electronic band structure calculated from the structural models for Cs_4_PbBr_6_ and Cs_4_PbI_6_ (PDF)
