# Supplementary material for: Structural Features and Optical Properties of All-Inorganic Zero-Dimensional Halides Cs4PbBr6–xIx Obtained by Mechanochemistry
Source: ACS Appl Mater Interfaces. 2023 Aug 18;15(34):40762–71. doi: 10.1021/acsami.3c07707 (PMC10472433; doi:10.1021/acsami.3c07707)
Supplement: Supplementary file 1 — am3c07707_si_001.pdf [file am3c07707_si_001.pdf]

SUPPORTING INFORMATION

Structural features and optical properties of all-inorganic zero-dimensional halides $Cs_4PbBr_{6-x}I_x$ obtained by mechanochemistry

Carmen Abia,[a,b] Carlos A. López[a,c], Javier Gainza[a], João Elias F. S. Rodrigues[d,e], Brenda Fragoso[f], Mateus M. Ferrer[f], Maria Teresa Fernández-Díaz,[b] François Fauth[d], José Luis Martínez[a], and José Antonio Alonso[a*]

[a]*Instituto de Ciencia de Materiales de Madrid, CSIC, Cantoblanco 28049 Madrid, Spain.*

[b]*Institut Laue Langevin, BP 156X, Grenoble, F-38042, France.*

[c]*INTEQUI, (UNSL-CONICET) and Facultad de Química, Bioquímica y Farmacia, UNSL, Almirante Brown 1455, 5700, San Luis, Argentina.*

[d]*CELLS–ALBA Synchrotron E-08290 Cerdanyola del Valles, Barcelona, Spain.*

[e]*European Synchrotron Radiation Facility (ESRF), 38000 Grenoble Cedex, France.*

[f]*CCAF, PPGCEM/CDTec, Federal University of Pelotas, 96010-610 Pelotas, Rio Grande do Sul, Brazil.*

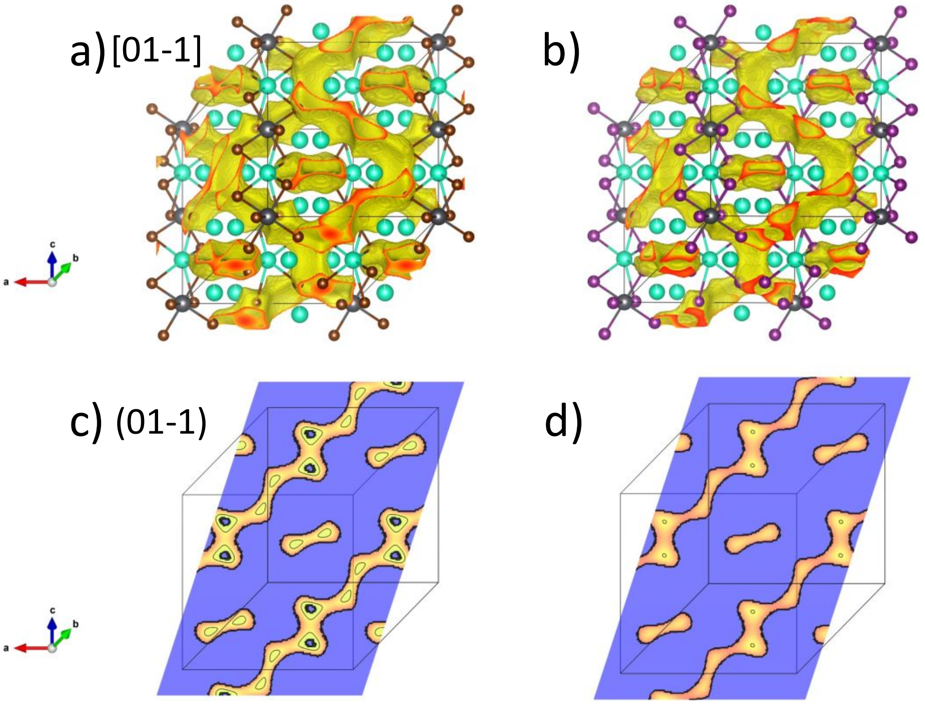

Figure S1. Isovalent surfaces (a and b) in Cs_4PbBr_6, showing the channels in the density planes (0 1-1), as represented in (c) and (d).

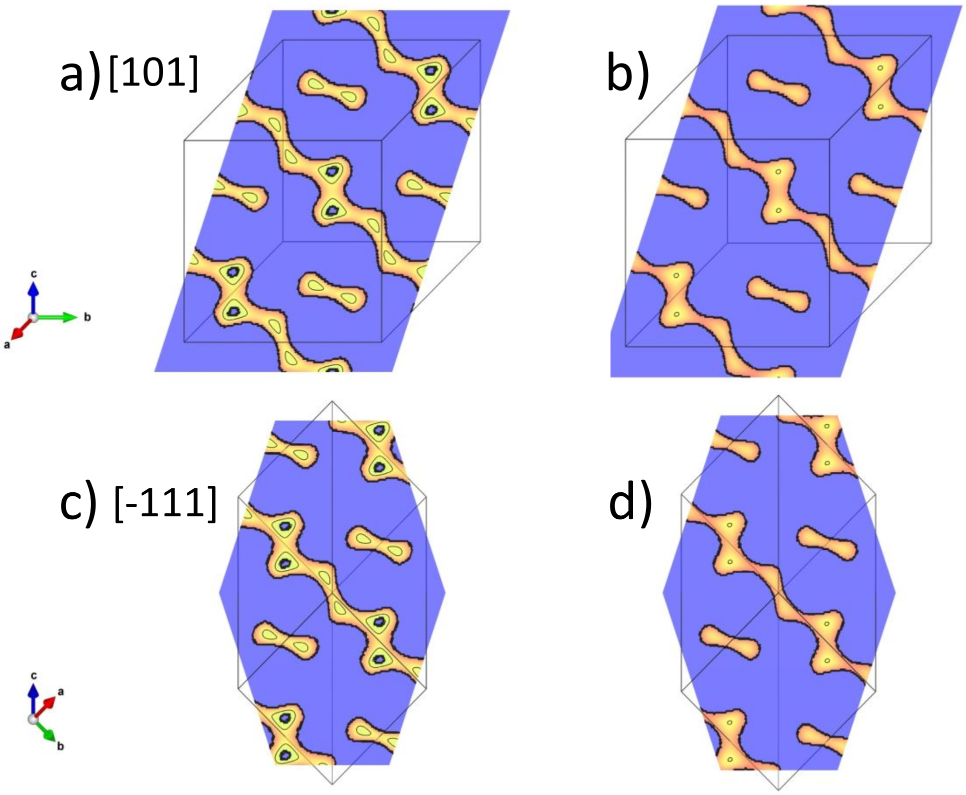

Figure S2. Additional isovalent planes for the diffusion of Br^- ions in Cs_4PbBr_6, corresponding to (a and b) [1 0 1] plane and (c and d) [-1-1-1] plane.

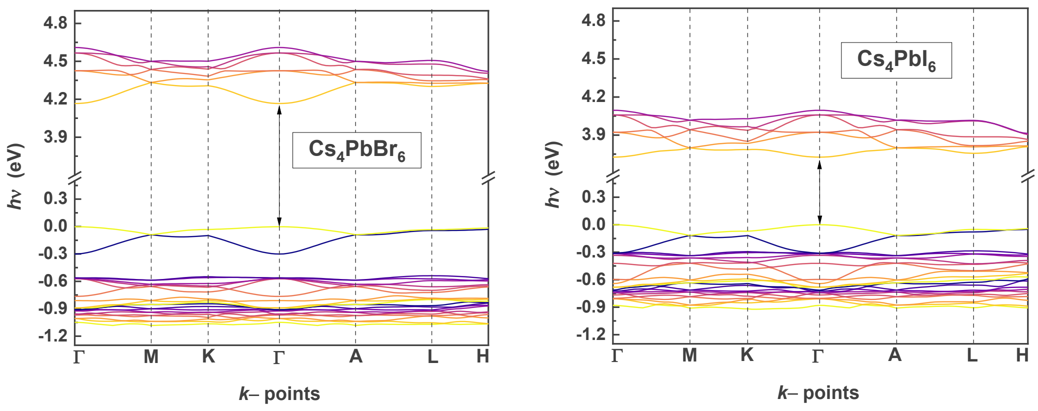

Figure S3. Electronic band structure calculated from the structural models for Cs_4PbBr_6 and Cs_4PbI_6. These structures unveiled that the bandgap is direct.
